# Prevalence and risk factors of SARS-CoV-2 antibody responses among healthcare workers (June 2020–November 2021)

**DOI:** 10.1093/eurpub/ckad093

**Published:** 2023-06-13

**Authors:** Maria Pilar Barrufet, Mateu Serra-Prat, Elisabet Palomera, Alícia Ruiz, Gemma Tapias, Noemí Montserrat, Nicolas Valladares, Francisco Javier Ruz, Mireia Bolívar-Prados, Pere Clavé

**Affiliations:** Infectious Disease Unit, Department of Internal Medicine, Hospital de Mataró, Consorci Sanitari del Maresme, Mataró, Barcelona, Spain; Research Unit, Fundació Salut del Consorci Sanitari del Maresme, Mataró, Barcelona, Spain; Centro de Investigación Biomédica en Red de Enfermedades Hepáticas y Digestivas (CIBERehd), Barcelona, Spain; Research Unit, Fundació Salut del Consorci Sanitari del Maresme, Mataró, Barcelona, Spain; Laboratori de Referència de Catalunya, Consorci Sanitari del Maresme, Mataró, Barcelona, Spain; Occupational Health Unit, Hospital de Mataró, Consorci Sanitari del Maresme, Mataró, Barcelona, Spain; Occupational Health Unit, Hospital de Mataró, Consorci Sanitari del Maresme, Mataró, Barcelona, Spain; Occupational Health Unit, Hospital de Mataró, Consorci Sanitari del Maresme, Mataró, Barcelona, Spain; Information Technology Department, Consorci Sanitari del Maresme, Mataró, Barcelona, Spain; Centro de Investigación Biomédica en Red de Enfermedades Hepáticas y Digestivas (CIBERehd), Barcelona, Spain; Drug Research Ethics Committee (CEIm), Consorci Sanitari del Maresme, Mataró, Barcelona, Spain; Centro de Investigación Biomédica en Red de Enfermedades Hepáticas y Digestivas (CIBERehd), Barcelona, Spain; Research and Academic Department, Hospital de Mataró, Mataró, Barcelona, Spain

## Abstract

**Background:**

To assess SARS-CoV-2 seroprevalence in healthcare workers (HCW) with sampling in June and October 2020 and April and November 2021.

**Methods:**

Observational and prospective study in 2455 HCW with serum sampling. Antibodies against SARS-CoV-2 nucleocapsid and occupational, social and health risk factors were assessed at each time point.

**Results:**

Seropositivity for SARS-CoV-2 in HCW increased from 11.8% in June 2020 to 28.4% in November 2021. Of those with a positive test in June 2020, 92.1% remained with a positive test, 6.7% had an indeterminate test and 1.1% had a negative test in November 2021. Non-diagnosed carriers represented 28.6% in June 2020 and 14.6% in November 2021. Nurses and nursing assistants showed the highest prevalence of seropositivity. Close contact (at home or in the hospital) with Covid-19 cases without protection and working in the frontline were the main risk factors. A total of 88.8% HCW were vaccinated, all with a positive serological response in April 2021, but levels of antibodies decreased about 65%, and two vaccinated persons presented a negative serological test against spike protein in November 2021. Levels of spike antibodies were higher in those vaccinated with Moderna compared with Pfizer and the percentage of antibody reduction was higher with Pfizer vaccine.

**Conclusions:**

This study shows that seroprevalence of SARS-CoV-2 antibodies among HCW doubled that of the general population and that protection both at the workplace and in the socio-familial field was associated with a lower risk of infection, which stabilized after vaccination.

## Introduction

The first confirmed case of COVID-19 in Spain was 31 January 2020. From then until November 2021, a total of 5 111 842 confirmed cases and 87 904 deaths from COVID-19 occurred in Spain.^1^ By the end of April 2021, 130 609 healthcare workers (HCW) had been diagnosed with COVID-19 in Spain.^2^ Several studies have evaluated the seroprevalence against SARS-Cov-2 in the specific population of HCW who were at greater risk of contracting and spreading the disease.^3–9^ A recent systematic review of SARS-CoV-2 seroprevalence studies in Europe included data on HCW and the community and showed a wide heterogeneity in the reported prevalence of seroconversions within and between these two populations.^10^ Regarding HCW, studies from Spain reported a seroprevalence very close to 10% between March and August 2020^3–5^ but some studies reported higher seroprevalences in other European countries.^8^ Community studies performed in Europe during the same period reported very heterogeneous results, ranging from 0.42% in Greece^11^ to 23% in Italy^12^ but, in general, lower seroprevalences in comparison to HCW.^10^ Results of a nationwide, population-based, seroepidemiological study in Spain (ENE-COVID), performed between April and June 2020, reported a seroprevalence of 5.2%, and a third of individuals who had developed SARS-Cov-2 antibodies remained asymptomatic.^13^ The data are quite different because populations, geographic areas, epidemic situations, study dates and serological techniques vary between studies.

It is essential to know the risk factors for HCW to contract COVID-19 in order to design effective preventive measures. Likewise, assessing the prevalence of non-diagnosed carriers is crucial in order to develop effective strategies to prevent the spread of the disease from healthcare centers. In June 2020, the Maresme Health Consortium (CSdM), a medical consortium that manages a general hospital, a subacute hospital, a nursing home and three primary care centers, launched a prospective study to monitor seroprevalence against SARS-CoV-2 among its workers. This study not only determined SARS-CoV-2 antibodies at several time points but also the rate of negativizations and the evolution of SARS-CoV-2 antibodies after vaccination.

The main objectives of this study were: (i) to assess the evolution of the prevalence of exposure to SARS-CoV-2 of the 2455 professionals working at CSdM, Catalonia, Spain, over time (June 2020–November 2021); (ii) to determine the prevalence of non-diagnosed carriers; (iii) to explore occupational, social and health risk factors associated with SARS-CoV-2 infection; and (iv) to monitor the antibody responses following mRNA vaccination.

## Methods

### Study design and population

This observational and prospective study followed the cohort of all 2455 active CSdM workers from June 2020 to November 2021. All of them were informed about the study and invited to participate by e-mail and by announcements on the CSdM website. Once workers gave their informed consent, they answered an electronic self-administered questionnaire on personal sociodemographic, clinical and labor characteristics through the CSdM website, and scheduled a blood extraction for serological tests. This procedure was carried out in June 2020, October 2020, April 2021 and November 2021. Participation rates in the seroprevalence study were 77.9%, 77.1%, 84.3% and 68.9% of the total target population, respectively. Most CSdM workers (88.8%) received two doses of vaccine from January to March 2021, all of them with mRNA vaccines (74.4% Moderna COVID-19 Vaccine, Moderna Biotech Spain, 25.6% Comirnaty BioNTech, Pfizer). A figure in Supplementary material gives the epidemiological time context, showing the number of confirmed cases of COVID-19 treated at Mataró hospital from March 2020 to January 2022, the various phases of the study and the vaccination periods (first, second and third doses). The study protocol was approved by the Ethics Committee (CEIm, CSdM 56/20). The study was conducted according to the principles and rules stated in the Declaration of Helsinki and following the regulations established by Spanish biomedical research law (LIB 14/2007), the Spanish law of protection of personal data (LOPD 3/2018) and Regulation (EU) 2016/679 of the EU Parliament and of the Council of 27th April 2016. The study was registered in the ClinicalTrials.gov website under code: NCT04425759.

### Data gathering

The self-administered electronic questionnaire on risk factors for COVID-19 included sociodemographic characteristics, health status, comorbidities and chronic medication, current symptoms and labor characteristics, which included exposure to COVID-19 patients and use of protective measures. A summary of the epidemiological survey is presented in Supplementary appendix S1. The venous blood samples obtained in June 2020 and October 2020 were sent to the laboratory for the following serological tests: (i) chemiluminescence Immunoassay (CLIA) (Elecsys Cobas, Roche Diagnostics) to detect SARS-CoV-2 nucleocapsid-protein antibodies and, for those positive, (ii) a qualitative enzyme-linked Immunosorbent Assay (ELISA) to differentiate IgG from IgM and IgA antibodies for SARS-CoV-2 (COVID-19 ELISA IgG G1032; COVID-19 ELISA IgM+IgA MA1032, Vircell Microbiologists). In the case of positive IgM+IgA, a nasopharyngeal swab was obtained for the real-time reverse transcriptase–polymerase chain reaction (TaqPath COVID-19 CE-IVD RT–PCR, Thermo Fisher Scientific, Pleasanton, CA, USA) to detect asymptomatic carriers of the virus and participants who had had COVID-19 and still harbored the virus. If the results of the total antibodies CLIA test were undetermined and ELISA IgM+IgA and IgG were also undetermined, the serology test was repeated 10 days later. The venous blood samples obtained in April and November 2021 were sent to the laboratory for the following serological tests: (i) the same CLIA to detect SARS-CoV-2 nucleocapsid-protein antibodies as in June and October 2020 and (ii) a test to quantify SARS-CoV-2 spike-protein antibodies (DiaSorin LIAISON, TrimericS IgG assay) to determine post-vaccine seroconversion. All serum aliquots were stored in a sample collection registered in the ‘Registro Nacional de Biobancos’ (https://biobancos.isciii.es/ListadoColecciones.aspx) under the code: C0006111. The data that support the findings of this study are not openly available due to confidentiality norms, but are available from the corresponding author upon reasonable request and Ethical Committee approval.

### Data analysis

Seroprevalence against SARS-CoV-2, expressed as percentages and 95% confidence intervals (95% CI), and the prevalence of non-diagnosed carriers in June 2020, October 2020, April 2021 and November 2021 were estimated. An individual was considered positive if CLIA was positive (independently of IgA/IgM and IgG results), negative if CLIA was negative, indeterminate if CLIA was indeterminate, and false positive if CLIA was positive and both IgA/IgM and IgG were negative. The range of measurement of IgG SARS-CoV-2 SPIKE-TRIMERIC was 4.81–2080 BAU/ml (measurements >33.8 BAU/ml were considered positive). Cross-sectional (June 2020) and longitudinal (June 2020–April 2021) exploratory analyses of the study factors associated with a positive serological test result were performed using the Chi-square test or Fisher exact test and bivariate and multivariate logistic regression analyses. All variables associated with a positive serological test in the bivariate analysis (*P*-values <0.10) were considered for the multivariate analysis using the stepwise method. Gender was also included in the multivariate model (although not reaching a *P*-value <0.10 in the bivariate analysis). Factors related to non-diagnosed carriers in June 2020 were also analyzed using the same above-mentioned statistical tests (Chi-square or Fisher exact test and logistic regression analysis). Immunological vaccination response was assessed by describing the prevalence of HCW with positive antibodies against SARS-CoV-2 spike protein (as a percentage and 95% CI) in April 2021 and November 2021. Levels of anti-spike protein antibodies were compared between April 21 and November 21 using the Wilcoxon test for paired data. As multiple comparisons were made, the Bonferroni correction was applied and statistical significance was established at a *P*-value <0.0014. Statistical analysis was performed with the IBM SPSS Statistics software (version 28.0.0.0).

## Results

### Description of the study sample

**Figure 1:**
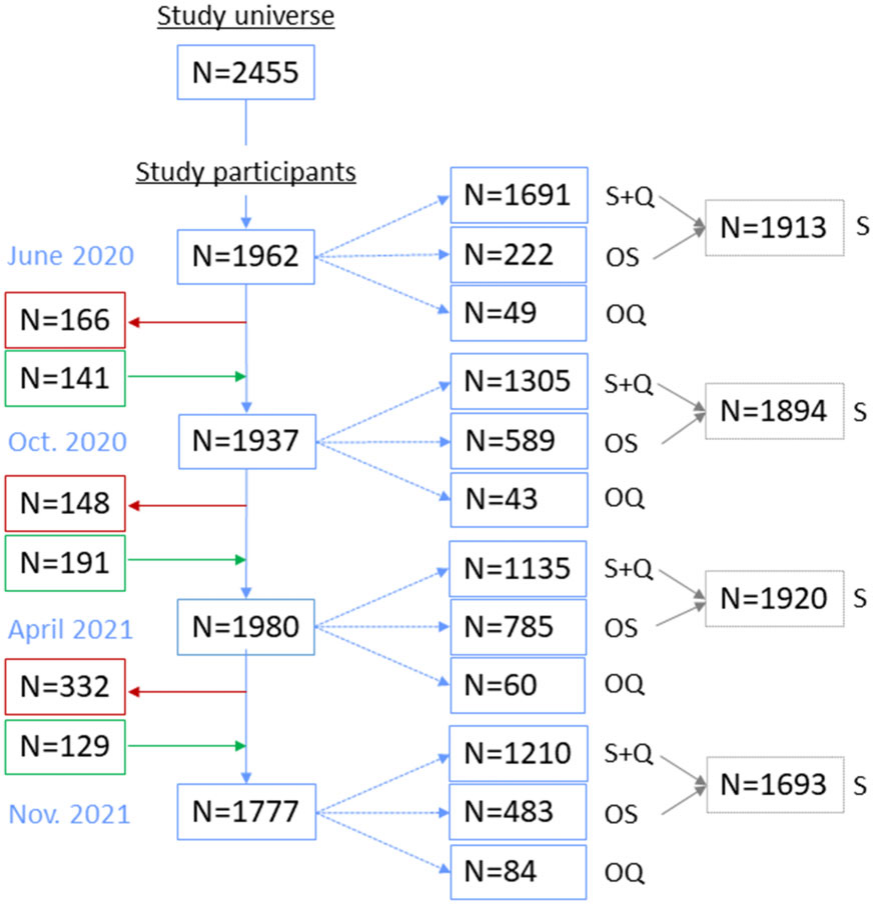
Flow chart of HCW participants in the seroprevalence study. S + Q, serological test + questionnaire; OS, only serological test; OQ, only questionnaire; S, serological test (with and without questionnaire)

Figure 1 presents the flow chart of participants in the study over time and indicates that the study cohort was dynamic with some workers leaving (retirements) and others entering (new incorporations) throughout the study period. At baseline (June 2020), the mean age of the study sample was 42.3 (±12.0) years (SD) and 1328 (78.5%) were women. Clinical, social and labor characteristics of the study sample are presented in table 1. Details on comorbidities and medication of the study sample are presented in Supplementary appendix S2.

**Table 1:** Factors associated with a positive serological test on June 20. Bivariate analysis

All sample		Negative serology	Positive serology	*P*-value	OR
*N* = 1691		*N* = 1486	*N* = 203		(95% CI)
Sociodemographic data					
Sex (% women)	1328 (78.5%)	1174 (79.0%)	152 (74.9%)	0.179	1.26 (0.90–1.76)
Age (±SD)	42.3 (12.0)	42.5 (12.1)	40.9 (11.5)	0.078	0.99 (0.98–1.00)
Living alone	141 (8.3%)	127 (8.5%)	14 (6.9%)	0.425	0.79 (0.48–1.41)
Living with health care workers	319 (18.9%)	270 (18.2%)	49 (24.1%)	0.042	1.43 (1.01–2.03)
Clinical data					
Hospitalization in the previous year	82 (4.8%)	65 (4.4%)	17 (8.4%)	0.013	1.99 (1.15–3.48)
Had a flu vaccine last season	424 (25.1%)	362 (24.4%)	62 (30.5%)	0.057	1.37 (0.99–1.88)
Had a tuberculosis vaccine at some time	354 (20.9%)	319 (21.5%)	35 (17.2%)	0.165	0.76 (0.52–1.12)
Had a pneumococcal vaccine	169 (10.0%)	148 (10.0%)	21 (10.3%)	0.864	1.04 (0.64–1.69)
Smoking habit					
Not current smoker	1334 (79.0%)	1149 (73.5%)	185 (91.1%)	<0.001	10.33 (0.20–0.55)
Current smoker	355 (21.0%)	337 (22.7%)	18 (8.9%)		
Been diagnosed with COVID 19	165 (9.8%)	20 (1.3%)	145 (71.4%)	<0.001	183.3 (107.2–313.2)
Labor data					
Occupational category					
1. Physician	324 (19.2%)	281 (18.9%)	43 (21.2%)	0.441	1.15 (0.80–1.65)
2. Nurse	472 (27.9%)	395 (26.6%)	76 (37.4%)	0.001	1.65 (1.22–2.25)
3. Nursing aid	326 (19.3%)	279 (18.8%)	46 (22.7%)	0.188	1.27 (0.89–1.81)
All HCW (1 + 2 + 3)	1120 (66.3%)	955 (64.3%)	165 (81.3%)	<0.001	2.41 (1.67–3.49)
4. Other HCW	132 (7.8%)	124 (8.3%)	8 (3.9%)	0.028	0.45 (0.22–0.94)
5. Patient transporters	34 (2.0%)	32 (2.2%)	2 (1.0%)	0.266	0.45 (0.11–1.91)
6. Administration staff	176 (10.4%)	159 (10.7%)	17 (8.4%)	0.309	0.76 (0.45–1.29)
7. Cleaning staff	102 (6.0%)	97 (6.5%)	5 (2.5%)	0.023	0.36 (0.15–0.90)
8. Other non-health care staff	115 (6.8%)	109 (7.3%)	6 (3.0%)	0.020	0.38 (0.17–0.89)
9. Steering committee	10 (0.6%)	10 (0.7%)	0	0.619	–
Normal workplace					
10. ICU	164 (9.7%)	151 (10.2%)	13 (6.4%)	0.090	0.61 (0.34–1.09)
11. Hospitalization	742 (43.9%)	632 (42.5%)	109 (53.7%)	0.003	1.57 (1.17–2.10)
12. Outpatient clinics	348 (20.6%)	309 (20.8%)	39 (19.2%)	0.601	0.91 (0.63–1.31)
13. Surgery	269 (15.9%)	250 (16.8%)	19 (9.4%)	0.006	0.51 (0.31–0.84)
14. Emergencies	390 (23.1%)	344 (23.1%)	45 (22.2%)	0.755	0.95 (0.67–1.35)
15. Day hospital	147 (8.7%)	132 (8.9%)	15 (7.4%)	0.479	0.82 (0.47–1.43)
16. Non-healthcare assistance	296 (17.5%)	274 (18.4%)	22 (10.8%)	0.008	0.54 (0.34–0.85)
17. Laboratory	84 (5.0%)	81 (5.5%)	3 (1.5%)	0.015	0.26 (0.08–0.83)
18. Pharmacy	67 (4.0%)	63 (4.2%)	4 (2.0%)	0.120	0.45 (0.16–1.26)
19. Radiology	109 (6.4%)	102 (6.9%)	7 (3.4%)	0.063	0.49 (0.22–1.06)
Frontline attention					
20. Was not frontline	711 (42.1%)	651 (43.8%)	60 (29.6%)	<0.001	11.39 (0.89–2.17)
21. Frontline, NOT feeling unprotected	299 (17.7%)	265 (17.8%)	34 (16.7%)		2.08 (1.49–2.90)
22. Frontline feeling unprotected	679 (40.2%)	570 (38.4%)	109 (53.7%)		
Kept a safe distance of 1.5 m in communal areas when not wearing a mask	1459 (86.3%)	1293 (87.0%)	164 (80.8%)	0.016	0.63 (0.43–0.92)
Been in close contact without protection with patients diagnosed with COVID at work.	388 (22.9%)	319 (21.5%)	68 (33.5%)	<0.001	1.84 (1.34–2.53)
Been in close contact without protection with workers diagnosed with COVID at work.	513 (30.3%)	445 (29.9%)	68 (33.5%)	0.302	1.18 (0.86–1.61)
Been in close contact without protection with family members diagnosed with COVID	89 (5.3%)	67 (4.5%)	22 (10.8%)	<0.001	2.57 (1.55–4.27)
Been in close contact with other people diagnosed with COVID outside work	72 (4.3%)	60 (4.0%)	12 (5.9%)	0.215	1.49 (0.79–2.83)

### Prevalence of positive antibodies against SARS-CoV-2 and non-diagnosed carriers

**Figure 2:**
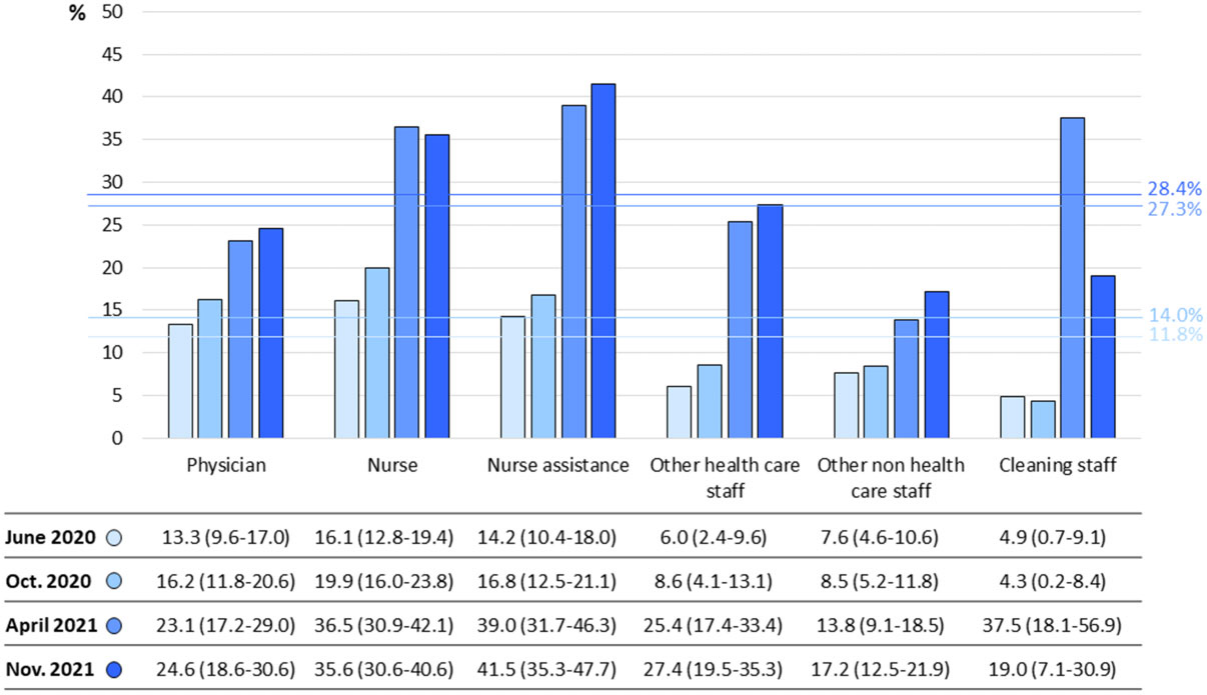
Prevalence of positive serological tests against the nucleocapsid proteins of SARS-Cov2 by professional categories at each time point

In June 2020, the prevalence of positive serological tests against the nucleocapsid proteins of SARS-CoV-2 was 11.8% (95% CI: 10.4–13.2%) of the whole study sample. In October 2020, April and November 2021, this prevalence was 14.0% (95% CI: 12.4–15.6%), 27.3% (95% CI: 25.3–29.3) and 28.4% (95% CI: 26.2–30.5), respectively. Figure 2 presents the prevalence of this positive serological test by professional categories at each time point. Of the workers with a positive CLIA test in June 2020, 92.1% still tested positive, 6.7% indeterminate and 1.1% tested negative in November 2021. Of the workers who were positive in June 2020, 10.8% were asymptomatic, 83.7% presented mild symptoms with no hospitalization, 3.9% presented moderate symptoms that required hospitalization and 1.5% presented severe symptoms that required ICU admission. Of those who were seropositive, the most prevalent symptoms were general unrest (65.0%), headache (56.2%), fatigue (54.7%), loss of smell (53.7%), loss of taste (53.2%), muscular pain (51.2%), diarrhea (31.0%) and dyspnea (27.6%). In June 2020, of the workers with positive serological tests against SARS-CoV2, 28.6% (95% CI: 26.4–30.8) had not been previously diagnosed with COVID-19 and did not know they had had it. This percentage was 26.3% in October 2020 (95% CI: 23.8–28.8), 22.4% in April 2021 (95% CI: 19.6–25.2) and 14.6% in November 2021 (95% CI: 12.1–17.1).

### Study factors associated with a positive serological test and for non-diagnosed carriers

Table 1 shows the socio-familial, clinical and labor characteristics associated with a positive serological test in June 2020 (cross-sectional analysis). According to the stepwise method, the multivariate logistic regression analysis showed the following variables independently associated with a positive serological test in June 2020: hospital admission for any reason in the previous year (OR = 2.25; 95% CI: 1.26–4.01; *P* = 0.006); close contact without protection with a family member diagnosed with COVID-19 (OR = 2.24; 95% CI: 1.33–3.79; *P* = 0.003); work as a physician, nurse or nurse assistance (OR = 2.14; 95% CI: 1.46–3.15; *P* < 0.001); work in the frontline feeling unprotected (OR = 1.57; 95% CI: 1.13–2.18; *P* = 0.007); close contact without protection with COVID-19 patients at work (OR = 1.51; 95% CI: 1.07–2.12; *P* = 0.019), male sex (OR = 1.44; 95% CI: 1.01–2.05; *P* = 0.044), and current smoker (0.32; 95% CI: 0.19–0.53; *P* < 0.001).

Independent baseline study factors associated with a positive serological test in April 2021 (longitudinal analysis) were: close contact without protection with a family member diagnosed with COVID-19 (OR = 1.93; 95% CI: 1.06–3.51; *P* = 0.033), to work as a physician, nurse or nurse assistance (OR = 1.46: 95% CI: 1.03–2.05; *P* = 0.032), to work in the frontline (OR = 2.32; 95% CI: 1.70–3.15; *P* < 0.001), current smoker (OR = 0.45; 95% CI: 0.30–0.68; *P* < 0.001) and age (OR = 0.99; 95% CI: 0.97–0.99; *P* = 0.026).

Among workers with positive serology, in June 2020 the main factors associated with undiagnosed SARS-Cov2 infection were age under 35 years (OR = 2.55; 95% CI: 1.37–4.76; *P* = 0.003), not having been vaccinated for TBC (OR = 0.36; 95% CI: 0.13–0.98; *P* = 0.046), working in the hospital (vs. other healthcare centers) (OR = 0.38; 95% CI: 0.15–0.95; *P* = 0.039), working in the ICU (OR = 0.22; 95% CI: 0.07–0.71; *P* = 0.012), working in the non-COVID pharmacy unit (OR = 0.10; 95% CI: 0.01–0.96; *P* = 0.046) and working in non-COVID units (OR = 0.09; 95% CI: 0.02–0.35; *P* < 0.001).

### Immunological response after mRNA vaccination

**Figure 3:**
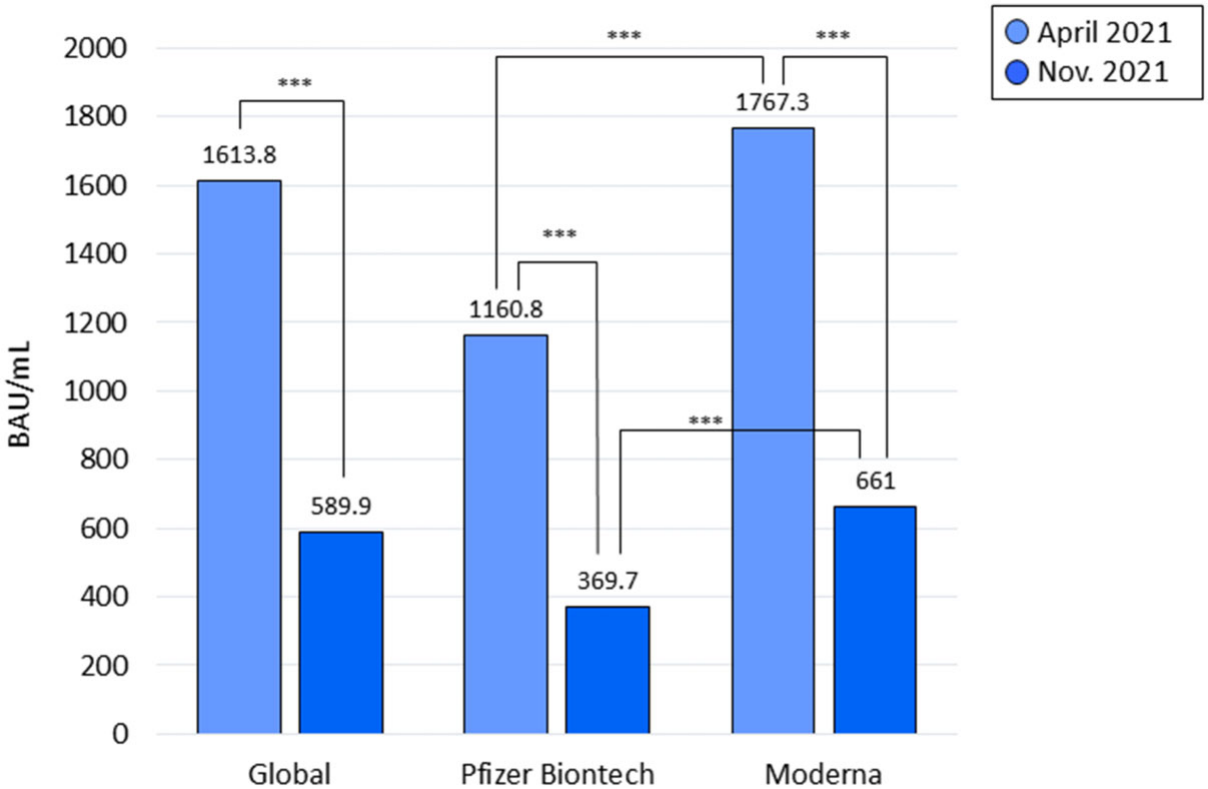
Mean levels of antibodies against spike protein according to the type of vaccine in vaccinated HCW who had not suffered COVID-19. ***P *<* 0.001

Mean (SD) levels of antibodies against spike protein were 1617.6 (555.0) BAU/ml among correctly vaccinated HCW who had not suffered COVID-19. All presented a positive serological test (>33.8 BAU/ml) against the spike protein of SARS-Cov2. Among vaccinated HCW who had not suffered COVID-19 since April 2021, mean levels of antibodies against spike protein decreased from 1613.8 BAU/ml (555.0) in April 2021 to 589.9 BAU/ml (523.1) in November 2021 (*P* < 0.001). Two vaccinated persons presented a negative serological test against spike protein in November 2021, both vaccinated with Pzifer. Figure 3 presents mean levels of antibodies against spike protein according to the type of vaccine in vaccinated HCW who had not suffered COVID-19. Levels of spike antibodies were higher in those vaccinated with Moderna compared with those vaccinated with Pfizer, both in April and November 2021 (*P* < 0.001 in both cases). The percentage of reduction in the level of antibodies was also higher in HCW that received the Pfizer vaccine compared with Moderna (65.1% vs. 48.7%, respectively, *P* = 0.005).

## Discussion

Main study results indicate that the prevalence of SARS-CoV-2 infection among HCW increased from 11.8% in June 2020 to 28.4% in November 2021 and affected, above all, HCW in direct contact with COVID-19 patients (nurses, nursing assistants and physicians). Results also indicate a higher risk of COVID-19 among nurses, nurse assistants and physicians compared with other HCW, and that adherence to protective measures may prevent SARS-CoV-2 infection. Approximately a quarter of professionals who were infected with SARS-CoV-2 had not been diagnosed, which would have favored the spread of the virus and some of the in-hospital outbreaks observed during the study period. The vaccine generated a positive immune response in all cases, which remained positive in 99.9% of cases 7 months later. However, anti-spike antibody levels decreased by ∼65% after 7 months, with still unknown clinical relevance. The growth of SARS-CoV-2 infection decreased sharply after the mRNA vaccines, which suggests their effectiveness.

Our results are similar to other seroprevalence studies carried out on HCW in Spain during the first wave of SARS-CoV-2 infection, in which seroprevalence ranged from 9.3% to 11.8%.^3–5^ A meta-analysis that included 25 seroprevalence studies worldwide for the year 2020 in HCW showed results that varied from 1.3% to 31.6% (mean 8%).^6^ The ENE-COVID was a nationwide population-based study aimed to estimate the seroprevalence of SARS-CoV-2 infection in Spain, which was carried out from 27 April to 11 May 2020.^13^ It showed a seroprevalence of 4.6% for the overall Spanish population and 6.8% for the population of the province of Barcelona.^4^ These data indicate that the seroprevalence of SARS-CoV-2 in HCW nearly doubled that of the general population. We have found only one longitudinal study that followed a cohort of workers during the first 2 years of the pandemia. It is a study with retrospective data collection from primary care electronic clinical notes in Barcelona (Spain), and shows an accumulated incidence of SARS-CoV-2 infection (from 20 March to 21 September) of 28% in HCW and of 14% in all other occupations.^7^ This result is consistent with our finding. Seroprevalence of CSdM HCW increased over time but became stabilized after April 2021, probably due to the vaccination program initiated in January and February 2021. The percentage of non-diagnosed HCW carriers was ∼25%, similar to that of other studies.^7–9^ These data raise the issue of systematic and periodic screening among HCW in an epidemic situation of high transmissibility to prevent spread among them and to preserve the functioning of the healthcare system.

The main factors associated with a positive serological test were to work as a physician, nurse or nurse assistant in the frontline with COVID-19 patients, and to be in close contact without protection with family members infected by SARS-Cov-2. The occupations with the highest overall seroprevalence were nurses and nursing assistants, who are the professional groups with the closest and most prolonged contact with patients. Likewise, unprotected contact with relatives with COVID-19 seems to have a similar effect, so it should be recommended not to relax protection and isolation measures at home. However, some differences were observed between study phases for the factors related to a positive serological test. Feeling unprotected working in the frontline or contact with patients without protection were factors related to a positive serological test in June 2020, but not in April 2021. At the beginning of the pandemic there was a shortage of appropriate material and protective equipment, which could explain why the feeling of being unprotected at work were associated with COVID-19 in June 2020 but not in April 2021. These results suggest the effectiveness of protective measures in the prevention of SARS-CoV-2 infection, but also highlight that this effectiveness is not absolute and that, despite such measures, HCW with the closest contact with COVID-19 patients had a more than 2-fold increase in the risk of SARS-CoV-2 infection over other HCW. A review showed that gender and inadequate/lack of protective personal equipment performing tracheal intubation were major risk factors of COVID-19 in HCW.^14^ On the other hand, tobacco consumption showed an independent association with SARS-CoV-2 infection with an OR of 0.45. This finding agrees with those published by other authors.^15–17^ However, it cannot be ruled out that this is a spurious association. There is controversy over the effect of smoking on SARS-CoV-2 infection, so further studies are needed to evaluate and clarify it. Furthermore, there is growing evidence that smokers have worse outcomes after contracting the virus than non-smokers,^18^ and a large-scale observational study suggested a causal effect of smoking on COVID-19 severity.^19^ More aged professionals were less likely to become infected, which may be explained by longer professional experience and better knowledge and use of preventive measures, which further reinforce the importance of knowledge, training, use and supervision of protection and prevention measures in the workplace.

Regarding SARS-CoV-2 vaccination, its acceptance by CSdM HCW was high (88%) and similar or slightly higher than that presented by other authors (between 70% and 80%).^20^ All vaccinated individuals presented positive anti-spike antibody levels, which is consistent with the results published by other authors.^21^ We observed that anti-spike antibody levels were higher among workers vaccinated with Moderna than those vaccinated with Pfizer, a difference that has also been reported in other studies.^22^ However, the clinical relevance of this difference is not known. Neutralizing antibody levels are predictive of greater immune protection from symptomatic infection,^23^ which suggests that higher levels may have some clinical relevance, especially in terms of maintaining longer-term protection as antibody levels decrease over time. Thus, at 7 months of vaccination, there was a 65% drop in anti-spike antibody levels in those vaccinated with Moderna and 68% in those vaccinated with Pfizer. Despite this, anti-spike antibodies remained positive in 99.9% of vaccinated individuals at 7 months follow up. These results agree with those presented by Doria et al.^24^ in relation to the Moderna vaccine. Finally, it should be noted that the seroprevalence of SARS-CoV-2 infection increased by 130% (from 11% to 27% of HCW) between June 2020 and April 2021 (before vaccination) but increased by <4% (from 27% to 28%) between April and November 2021 (after vaccination), which strongly suggests the effectiveness of mRNA vaccines.^25^

The relatively large sample size, high participation rate in all phases of the study with four blood samples over time, high vaccination acceptance and follow up of the study cohort for 17 months (6–7 months before and 10–11 months after vaccination) are important strengths of the study. However, it has some limitations. The fact that the study had an open cohort (with entrances and exits) made it possible to obtain an accurate picture of the epidemiological characteristics of the pandemic among CSdM HCW in real life, but made it difficult to assess risk factors that showed changes over time. Moreover, this is not a clinical trial but an observational study, so it is not the best design to assess the efficacy of a vaccine but it allowed us to assess its effectiveness in terms of immunological response in real conditions. The epidemic has also undergone changes over time, with outbreaks and virus mutations with variants with different clinical and epidemiological characteristics. We have presented epidemiological data in the context of the epidemic and its various outbreaks in our territory until the appearance of the omicron variant, which has radically changed the clinical features of COVID-19. With omicron, a new scenario arises in which epidemiological surveillance of new variants, new vaccinations or booster doses of vaccine, protection of the most vulnerable (such as aged or immunocompromised) or the search for new effective oral antivirals are some of the main challenges to face. Nevertheless, continuing to monitor the evolution of the immunological response against SARS-CoV-2 among HCW is important from the occupational health point of view and will be useful to assess immunological response protection against COVID-19.

## Supplementary Material

ckad093_Supplementary_Data

## Data Availability

The data that support the findings of this study are not openly available due to confidentiality norms, but are available from the corresponding author upon reasonable request and Ethical Committee approval. Seroprevalence of SARS-CoV-2 antibodies among HCW doubled that of the general population. Protection both at the workplace and in the socio-familial field is associated with a lower risk of infection. All vaccinated HCW showed a positive serological response, but levels of anti-spike antibodies decreased about 65% 7 months later.
