# Supplementary material for: Prevalence and risk factors of SARS-CoV-2 antibody responses among healthcare workers (June 2020–November 2021)
Source: Eur J Public Health. 2023 Jun 13;33(5):923–9. doi: 10.1093/eurpub/ckad093 (PMC10567251; doi:10.1093/eurpub/ckad093)
Supplement: ckad093_Supplementary_Data [file ckad093_supplementary_data.zip › ckad093_Supplementary_Data/ejph-2022-11-om-0549-File005.pdf]

Supplementary Appendix 1. Summary of the epidemiological survey.

**Seroprevalence study of SARS-CoV-2 antibodies in healthcare workers of the Maresme Health Consortium**
**Study identification (NID):**
**BASAL VISIT (Date)**
**I) Your workplace**
1. Professional category:<br>2. What is your usual Work Center?<br>3. What is your Service / Unit / Department:<br>4. What is your usual work area (allows multiple answers):<br>5. What is your work schedule (allows multiple answers):<br>6. Have you been to any other workplace than usual?
**II) Exposure to SARS-CoV-2**
7. During the COVID period (March 1 to the date of the survey) did you do face-to-face work at the CSdM?<br>8. Have you worked in COVID units?<br>If so, where?<br>9. Have you been in the first line of care (direct contact with COVID patients)? If yes, specify days<br>If so, have you had any unintended incidents with PPE? If so, which:<br>10. Have you ever felt unprotected with the protective equipment available, being in the frontline?<br>11. Have you worked in non-COVID units?<br>12. In common areas when you were not wearing a mask did you keep the safety distance of 1.5 meters?<br>13. Have you been overworked?<br>14. Have you had COVID prophylaxis? No / Yes<br>If yes, which:<br>15. Have you been diagnosed with COVID infection 19? If so, how were you diagnosed?<br>16. Have you been diagnosed with pneumonia:<br>17. Date of the test (the first to be positive)<br>18. Have you been in solitary confinement?<br>19. Have you been admitted to a hospital?<br>20. Have you been admitted to an ICU?<br>21. Have you been admitted to a health center?<br>22. Have you taken treatment for COVID?
**IV) Your health**
23. Background:<br>23.1- HTA<br>23.2- Diabetes:<br>23.3- Cardiovascular disease (heart, brain):<br>23.4- Chronic respiratory disease<br>23.5- Neurological or neurodegenerative diseases:<br>23.6- Anxiety / depression / other psychiatric illnesses:<br>23.7- Immunosuppression:<br>23.8- Immunosuppressive treatment:<br>23.9- Autoimmune disease:<br>23.10- Musculoskeletal diseases (including osteoarthritis):<br>23.11- Arthritis:<br>23.12- Active cancer in the last 5 years:

23.13- Liver disease
23.14- Kidney disease:
23.15- Pregnancy:
23.16- Breastfeeding:
23.17- Has menopause started?
23.18- In the last year, were you admitted to the hospital for some reason:

24. Chronic medication:
    24.1- Antihypertensives:
    24.2- Antidiabetics:
    24.3- Antacids (Omeprazole or similar):
    24.4- Cholesterol medications:
    24.5- Anti-inflammatory drugs (ibuprofen or similar):
    24.6- Antipyretics or analgesics (paracetamol):
    24.7- Inhaled corticosteroids:
25. Toxic habits
    25.1- Smoking habit:
26. Vaccination:
    26.1- Have you been vaccinated in the last season for the flu?
    26.2- Have you ever been vaccinated against TB?
    26.3- Have you ever been vaccinated against Pneumococcus?

V) Sociodemographic factors

27. Sex:
28. Age:
29. Weight:
30. Size:
31. Municipality of residence:
32. Family situation:
33. Number of people living in your home:
34. Do you live with people in essential services?
35. Have you been in close contact with patients diagnosed with COVID without protection at work?
36. Have you been in close contact with workers diagnosed with COVID without protection at work?
37. Have you been in close contact, unprotected, with relatives diagnosed with COVID?
38. Have you been in close contact with other people diagnosed with COVID outside of work?
