# Supplementary material for: Prevalence and risk factors of SARS-CoV-2 antibody responses among healthcare workers (June 2020–November 2021)
Source: Eur J Public Health. 2023 Jun 13;33(5):923–9. doi: 10.1093/eurpub/ckad093 (PMC10567251; doi:10.1093/eurpub/ckad093)
Supplement: ckad093_Supplementary_Data [file ckad093_supplementary_data.zip › ckad093_Supplementary_Data/ejph-2022-11-om-0549-File006.pdf]

**Supplementary Appendix 2.** Clinical characteristics of study sample.

| Clinical data | |
|---|---|
| **BMI** | 24.8 (4.5) |
| **Comorbidities** | |
| • High blood pressure | 124 (7.3%) |
| • Diabetes | 26 (1.5%) |
| • Cardiovascular disease (heart, blood vessel) | 42 (2.5%) |
| • Chronic respiratory disease | 107 (6.3%) |
| • Neurologic or neurodegenerative disease | 17 (1.0%) |
| • Anxiety/depression/other psychiatric diseases | 123 (7.3%) |
| • Immunosuppression | 14 (0.8%) |
| • Immunosuppressive treatment | 16 (0.9%) |
| • Auto-immune disease | 73 (4.3%) |
| • Musculoskeletal disorders (including arthrosis) | 159 (9.4%) |
| • Arthritis | 35 (2.1%) |
| • Cancer, active in the previous 5 years | 18 (1.1%) |
| • Liver disease | 24 (1.4%) |
| • Kidney disease | 8 (0.5%) |
| **Chronic medication** | |
| • Antihypertensive | 123 (7.3%) |
| • Antidiabetic | 23 (1.4%) |
| • Antiacids (Omeprazole or similar) | 119 (7.0%) |
| • Medication for cholesterol | 86 (5.1%) |
| • Anti-inflammatory (ibuprofen or similar) | 158 (9.3%) |
| • Antipyretic or analgesics (paracetamol) | 159 (9.4%) |
| • Inhaled corticosteroids | 76 (4.5%) |
